# Clinical features and treatment of “Non-dislocated hyperextension tibial plateau fracture”

**DOI:** 10.1186/s13018-020-01806-3

**Published:** 2020-07-29

**Authors:** Jiang Liangjun, Zheng Qiang, Pan Zhijun, Zhu Hanxiao, Chen Erman

**Affiliations:** The orthopedics department, 2nd affiliated hospital of medical college of Zhejiang university, The jiefang road 88#, Hangzhou, Zhejiang China

**Keywords:** Non-dislocated hyperextension tibial plateau fracture, Tibial plateau posterior slope angle, Ligament injury, Knee joint stability

## Abstract

**Background:**

To explore the epidemiological characteristics, clinical characteristics, treatment strategies, and clinical results of non-dislocated hyperextension tibial plateau fracture.

**Method:**

A total of 25 cases of non-dislocated hyperextension tibial plateau fracture patients were collected (12 males and 13 females), aged 27–79 years. Preoperative tibial plateau posterior slope angle was − 10~0° (average − 5.2°). Preoperative MRI showed 5 cases of MCL injury, 3 cases of PLC complex injury, and 2 cases of PLC + PCL injury. The change of tibial plateau posterior slope angle was more than 10° in patients with ligament injury, and the patients with a tibial plateau posterior slope angle change less than 10° had no ligament injury; 6 patients with simple column fracture had a ligament injury, 2 patients with bilateral column fracture had a ligament injury, and 2 patients with three column fracture had a ligament injury.

**Results:**

Patients were followed up for 12–24 months (average 16.4 months). The operative time was 65–180 min (average 124 min), and the blood loss was 20–200 ml (average 106 ml). The plate was placed on the anterior part of tibial plateau. Evaluation of postoperative fracture reduction was as follows: 20 cases reached anatomic reduction, 5 cases reached good reduction (between 2 and 5 mm articular surface collapse), and the excellent rate of fracture reduction was 100%. The fracture healing time was 3–6 months (average 3.3 months). The postoperative knee Rasmussen score was 18–29 (average 24.9), and the postoperative knee joint mobility was 90–130° (average 118°). Two patients suffered superficial infection.

**Conclusions:**

The main imaging characteristic of “non-dislocated hyperextension tibial plateau fracture” is the change of tibial plateau posterior slope angle. The injury of single anteromedial column/anterolateral column fracture is easy to combine with “diagonal” injury, and when the tPSA changes more than 10°, it is easy to be combined with ligament injury. By reducing the joint articular surface and lower limb force line, repairing the soft tissue structure, and reconstructing the knee joint stability, we can get satisfactory results.

**Trial registration:**

It was a retrospective study. This study was consistent with the ethical standards of the Second Affiliated Hospital of Zhejiang University Medical College and was approved by the hospital ethics committee and the trial registration number of our hospital was 20180145.

## Background

More and more attention is paid to the tibial plateau fracture injury mechanism at present, it is clear that tibial plateau fracture injury mechanism can carry out targeted reduction and treatment of plateau fracture, and the existing mechanism of injury speculation is generally based on Luo CF’s three-column theoretical classification [1, 2]. According to the three-column theoretical classification, the tibial plateau fracture injury mechanism can be divided into two categories on sagittal plane, such as extension type injury when the posterior slope angle of tibial plateau (pTSA) decreases, and flexion type injury when the posterior slope angle of tibial plateau (pTSA) increases. But in practice, these two types of classification cannot completely classify the plateau fracture. Such as the tibial plateau fracture type with knee dislocation which classified in Moore classification [3] does not have a significant posterior slope angle (pTSA) change and could not determine the injury mechanism for such fractures according to the posterior slope angle (pTSA) of the tibial plateau. Some tibial plateau fractures are caused by axial violence when the knee joint is hyperextended, resulting in the collapse of the anterior tibial plateau and the posterior soft tissue damage. This type of injury is relatively special, and the injury mechanism and treatment plan are different from other type plateau fractures, which are now generally referred to as “hyperextension tibial plateau fracture”.

Hyperextension tibial plateau fracture is rare in clinical practice. In 2001, Chiba et al. [4] firstly described 12 cases of “posterolateral knee injuries with anterior medial compression fractures of the tibial plateau.” In 2009, Jae HY et al. [5] reported one case report about “anterior compression of the tibial plateau.” Reza Firoozabadi et al. [6] reported 25 cases of “hyperextension varus bicondylar tibial plateau fracture (HVBT)” in 2016. However, there is currently no clear definition of hyperextension tibial plateau fracture, and the previous literature reports mostly describe one of these types of features. Because of the locking effect of the knee joint in the hyperextended position, the collapse of the anterior plateau often happens, which will cause the posterior slope angle to become smaller or even negative. It is easy to cause the posterior soft tissue structure damage or “diagonal” injury during the injury, which leads to joint instability when the knee joint is straightened. Therefore, surgery is often required. Due to the lack of attention to the mechanism and the cause of instability, it is often easy to ignore the repair of damaged soft tissue in such fractures, which may lead to joint instability in the later stage. At present, it is still difficult to treat such fracture, and the current treatment plan is not unified.

We retrospectively analyzed patients with hyperextension tibial plateau fracture treated in our hospital in recent years. Due to the injury mechanism greatly differenced from dislocated knee joint injury, the fracture-dislocation type tibial plateau fracture was excluded. By collection of clinical data of patients with non-dislocated hyperextension tibial plateau fracture, we hope to clarify (1) the clinical diagnostic criteria of non-dislocated hyperextension tibial plateau fracture; (2) the bone and soft tissue damage evaluation in non-dislocated hyperextension tibial plateau fracture; and (3) clinical treatment plan and prognosis of non-dislocated hyperextension tibial plateau fracture.

## Methods

The patient inclusion criteria were as follows: (1) tibial plateau pTSA change (unilateral/bilateral) with anterior compression of the bone mass, (2) injury to surgery time less than 3 weeks, and (3) patient follow-up time greater than 1 year. The exclusion criteria were as follows: (1) the fracture-dislocation type tibial plateau fractures (Moore classification), (2) evaluation of postoperative function severely affected by psychiatric or other cognitive disorders, (3) pathological fractures, and (4) open tibial plateau fractures.

This study included a total of 25 patients (12 males and 13 females) in our hospital from January 2018 to December 2018, aged 27–79 years (average 51 years). Causes of injury were as follows: 18 cases of traffic accidents, 2 cases of heavy crushes, and 5 cases of falls. Schatzker classification of tibial plateau fractures were as follows: 4 cases of type II, 5 cases of type IV, 13 cases of type V, and 3 cases of type VI; three-column theory classification were as follows: 4 cases of simple lateral column, 5 cases of simple medial column, 7 cases of medial column + lateral columns, and 9 cases of three columns. The tibial plateau posterior slope angle was − 10 to 0° before surgery (average − 5.2°) (Table 1).

**Table 1:** Patient baseline characteristics

No	Gender	Age	Injury cause	Schatzker classification	Three-column theory classification	Preoperative pTSA	pTSA change	MRI finding injury
1	Female	56	Traffic accident	II	Lateral column	− 8	14	MCL + mensci
2	Female	57	Traffic accident	V	Lateral+medial columns	− 10	20	MCL + mensci
3	Male	41	Traffic accident	V	Three columns	− 5	9	/
4	Male	37	Traffic accident	V	Lateral+medial columns	− 5	8	/
5	Female	31	Traffic accident	V	Three columns	0	5	/
6	Male	31	Traffic accident	VI	Three columns	− 3	7	/
7	Female	58	Traffic accident	V	Three columns	− 5	8	Mensci
8	Female	79	Traffic accident	V	Lateral+medial columns	− 8	20	MCL + mensci
9	Female	27	Traffic accident	V	Lateral+medial columns	− 5	9	/
10	Male	36	Fall	IV	Medial column	− 10	18	PCL + PLC + mensci
11	Male	41	Heavy crush	II	Lateral column	0	4	/
12	Female	51	Traffic accident	II	Lateral column	0	5	/
13	Male	63	Traffic accident	V	Lateral+medial columns	0	5	Mensci
14	Female	48	Traffic accident	V	Lateral+medial columns	− 8	14	MCL
15	Male	64	Heavy crush	VI	Three columns	− 5	10	/
16	Male	74	Traffic accident	IV	Medial column	− 10	20	PCL + PLC + mensci
17	Male	37	Fall	V	Three columns	− 10	18	PLC + mensci
18	Male	61	Fall	VI	Three columns	− 5	10	/
19	Female	31	Traffic accident	IV	Medial column	− 5	10	/
20	Female	69	Traffic accident	IV	Medial column	− 4	8	/
21	Female	61	Traffic accident	V	Three columns	− 6	12	PLC + mensci
22	Female	64	Traffic accident	II	Lateral column	− 5	12	MCL
23	Female	68	Fall	IV	Medial column	− 8	15	PLC + mensci
24	Male	46	Fall	V	Three columns	0	5	/
25	Male	56	Traffic accident	V	Lateral+medial columns	− 5	10	/

In the MRI examination, 5 cases were combined with MCL injury, 3 cases were combined with PLC complex injury, 2 cases were combined with PLC + PCL injury, and 10 cases were combined with meniscus injury. The tibial pTSA in patients with ligament injury change was greater than 10°. If the fracture was single-column fracture, we determined the pTSA change compared with the normal plateau column pTSA. If the fracture was bilateral column fracture, we determined the pTSA change compared with the patient’s contralateral limb’s plateau pTSA by x-ray. In these cases, 5 patients had a change of pTSA greater than 15°, and 5 patients had a change of pTSA greater than 10°, but the patients with a change of pTSA less than 10° had no combined ligament injury; 6 patients with only lateral or medial column fracture, 2 patients with medial and lateral column fractures, and 2 patients with three-column fractures were combined with ligament injury. One patient had popliteal artery injury, and this patient showed disappearance of dorsal foot artery pulse; 2 patients had common peroneal nerve injury, and these patients showed loss of ankle dorsiflexion and dorsalis pedis hypoesthesia; 2 patients had osteofascial compartment syndrome, and these two patients showed obvious edema of the affected lower limbs, the presence of toe stretch pain, and movement disorders.

This study complied with the ethics standards of the Second Affiliated Hospital of Zhejiang University School of Medicine and was approved by the hospital ethics committee.

### Preoperative plan

The affected limb was fixed with plaster or calcaneal bone traction. X-ray, CT, and MRI examinations were usually performed. We did the MRI before calcaneal bone traction. Surgical treatment was performed after edema of the affected limb subsided. For patients with popliteal artery injury or compartment syndrome, emergency surgery was performed. Fracture treatment was performed in the second stage. According to the preoperative examination, the fracture characteristics were clarified, and the surgical approach and fixation methods were formulated.

### Surgical treatment

The patient was supine on a fluoroscopic operating table under general anesthesia, and a tourniquet was placed on the affected limb. According to the preoperative examination, the corresponding surgical approach and treatment strategy were adopted.

For the anterior medial platform fracture, a 10-cm long medial longitudinal incision of the knee joint is made, and the skin, subcutaneous, and deep fascia were separated layer by layer. The medial meniscus margin was placed with the suture line for traction and lifted proximally to show the medial tibial plateau articular surface. Then, we directly reduced the collapsed articular surface, restored to normal posterior slope angle, gave bone graft support to bone defects, and made Kirschner wire temporary fixation. After satisfactory reduction, the anatomical locking plate was used to fix the medial plateau. After the fracture was fixed, the stability of the knee joint was checked by the anterior and posterior drawer tests, knee flexion 0° and 30° varus or valgus stress tests, and dial tests. It was suggested that the posterolateral structure of the knee joint should be repaired in the presence of knee instability after fracture reduction.

For anterolateral plateau fractures, we placed the affected limb in a slightly flexed position and made a 10–15 cm arc incision centering on the Gerdy nodule. After skin incision, we splitted the iliotibial tract along the fiber direction in the middle site and made a sharp dissection from the Gerdy’s nodule. The lateral anterior articular capsule was incised transversely, and sutures were placed on the edge of the lateral meniscus for traction and lifted proximally to expose the lateral tibial plateau articular surface. The collapsed articular surface was lifted up under direct vision to restore the normal pTSA. The bone graft was given, and the Kirschner wire was temporarily fixed. After satisfactory reduction, the anatomical locking plate was used to fix the lateral plateau. After that, the stability of the knee joint was checked by physical tests as described above; if it was accompanied by instability of the knee joint, the medial structure of the knee would be repaired.

For medial and lateral plateau fractures, surgery was performed using medial and lateral incisions, as described above.

For medial structural injury, the knee joint was still unstable after the lateral plateau fracture reduction, and fixation was completed during the operation. Then, a 10-cm long medial incision was made. The damaged tissue was sutured during the operation, and the edge of the joint capsule was sutured with a suture anchor (Arthrex, USA); the medial collateral ligament attachment tear was sutured with a suture anchor (Arthrex, USA); the body injury of medial collateral ligament was repair with a direct suture. For meniscus damage, 2–0 absorbable sutures were used for suture repair. If sutures could not be sutured, meniscus plasty was performed.

For the posterolateral structural injury, the knee joint was still unstable after the reduction and fixation of the medial plateau fracture during the operation. Then, another 10–15 cm long lateral incision was made to show the iliotibial bundle, the biceps femoris tendon, and the fibula head. The damaged tissue was sutured during the operation, and the edge of the joint capsule was sutured with a suture anchor (Arthrex, USA); the little fibula head fracture with avulsion at the lateral collateral ligament attachment point was fixed with a suture anchor; the large fibula head fracture with a large avulsion at the attachment point of the biceps femoris tendon was fixed with a wire tension band. For meniscus damage, 2–0 absorbable sutures were used for suture repair. If sutures could not be sutured, meniscus plasty was performed.

After all repairs were completed, the physical tests were performed to check the stability of the knee joint again. The operation was terminated after confirming that there was no knee instability.

### Postoperative management

Antibiotics were used for 2 days after surgery to prevent infection, and other treatment such as pain killer and anticoagulation were also used. On the first day after surgery, bed joint function activities were started. After 2 weeks, partial weight-bearing functional training was allowed under the guidance of doctor.

### Follow-up and evaluation criteria

Regular follow-up was performed in 1, 2, 3, 6, 9, and 12 months after operation. The knee x-ray examination was performed every time, the knee CT examination was performed when necessary, and we checked the joint pain and joint mobility. We summarized the operation time, intraoperative blood loss, fracture healing time, fracture reduction, knee function score, knee mobility, and postoperative complications. Fracture healing time was judged according to clinical standards and imaging standards, Rasmussen scoring system [7] was used to judge knee joint function, and Biggi F [8] method was used to evaluate fracture reduction.

### Statistical analysis

The knee Rasmussen score and knee range motion were compared between no ligament injury patients and ligament injury patients by the independent *t* test. We used ROC curve (receiver operator characteristic curve) analysis to find the best cutoff value of pTSA degree change connected with the ligament injury. *P* < 0.05 was considered significant. The SPSS software (22nd edition, SPSS, Chicago) was used to make statistical analyze.

## Result

### General condition of the patients

**Fig. 1:**
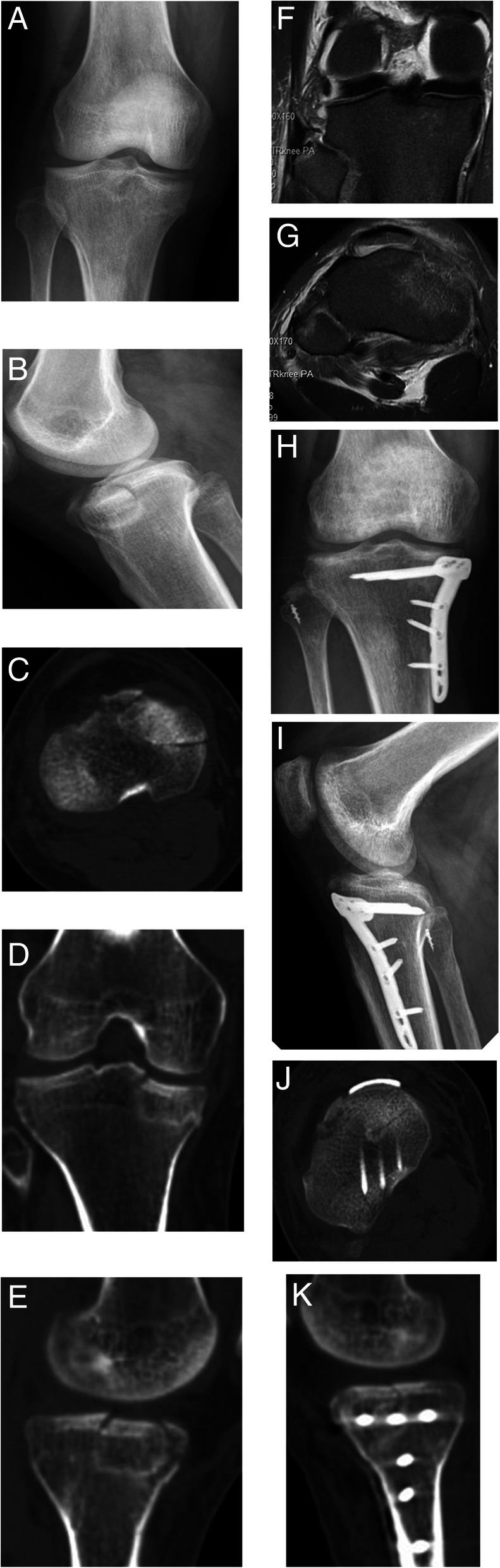
M, 36Y, anterior medial tibial plateau fracture with PLC injury. **a**, **b** Preoperative x-ray shows anteromedial tibial plateau fracture. **c**–**e** Preoperative CT shows collapse of anteromedial tibial plateau with significant change of pTSA. **f**, **g** Preoperative MRI showed posterolateral angle injury and partial tear of lateral collateral ligament at fibula. **h**–**k** Postoperative x-ray and CT showed the anatomical tibial plateau fracture reduction, and the lateral collateral ligament was repaired with a suture anchor

**Fig. 2:**
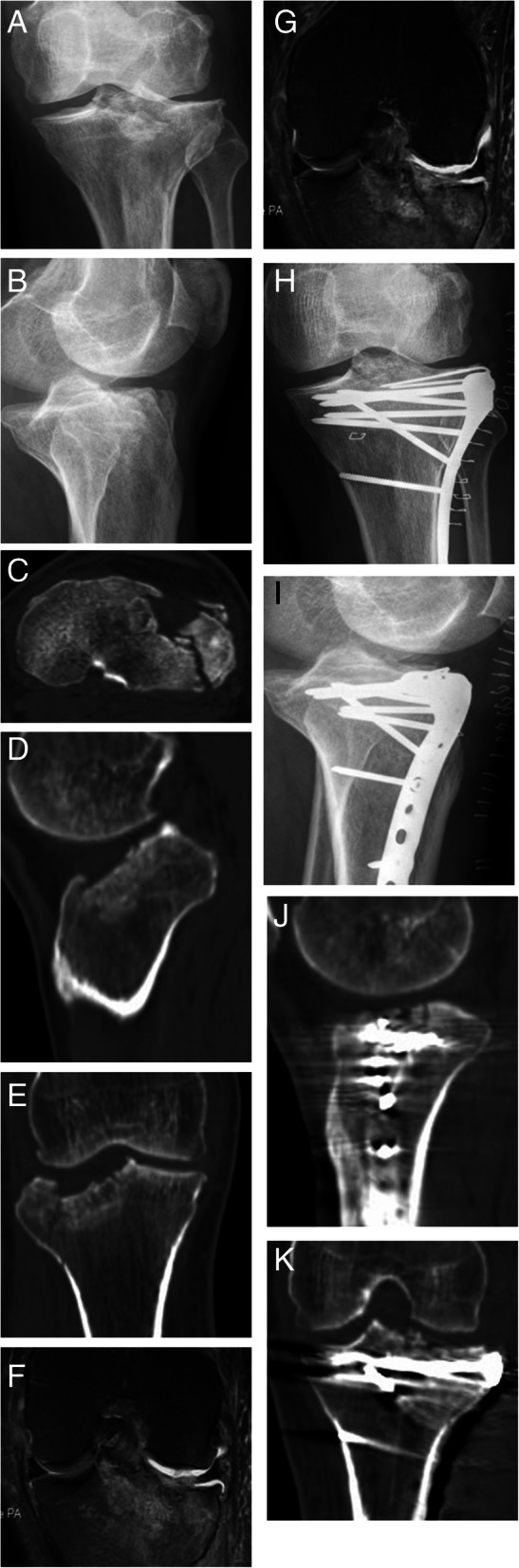
F, 56Y, anterior, and lateral tibial plateau fractures with MCL injury. **a**, **b** Preoperative x-rays of the tibial anterolateral plateau fracture.**c**–**e**. Preoperative CT shows anterolateral tibial plateau collapse and splitting, significant change in pTSA. **f**, **g** Preoperative MRI shows medial collateral ligament damage. **h**–**k** Postoperative x-ray and CT show an anatomical reduction of tibial plateau fracture

**Fig. 3:**
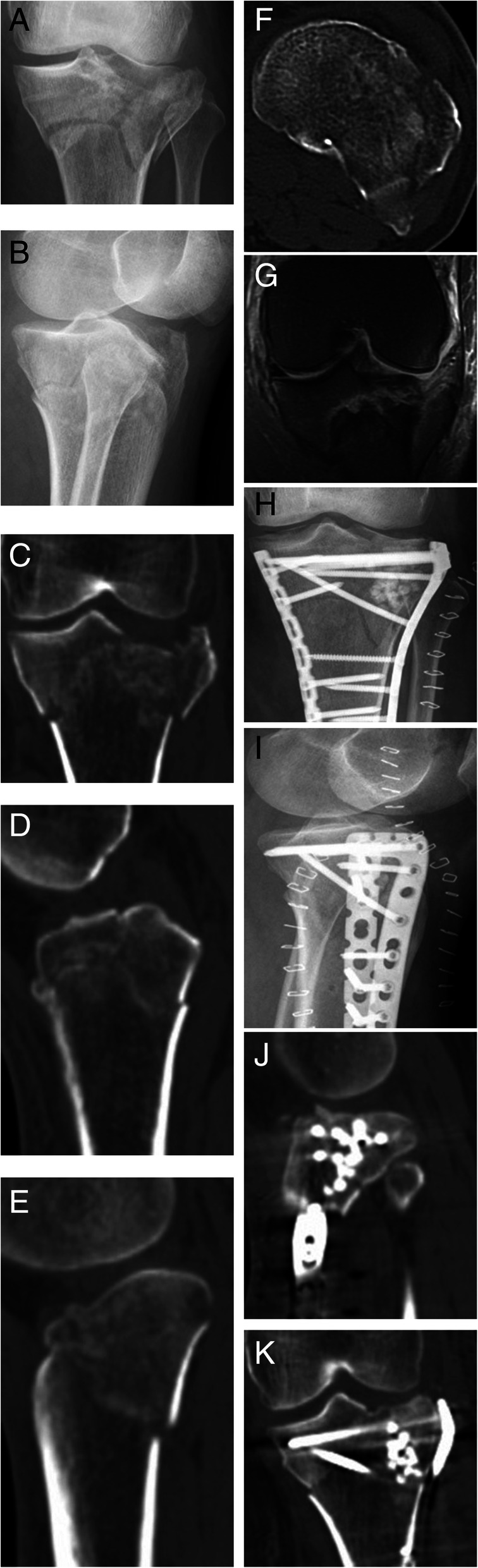
F, 57Y, medial, and lateral plateau fractures with MCL injury. **a**, **b** Preoperative x-ray shows the medial and lateral tibial plateau fractures and significant change in pTSA. **c**–**f** Preoperative CT scans show collapse and splitting of the bilateral plateau fractures and the anteverted plateau. **g** Preoperative MRI shows medial collateral ligament injury. **h**–**k** Postoperative x-rays and CT show anatomical fracture reduction and the artificial bone graft

A total of 25 patients were collected in the study. The follow-up time was 12–24 months (average 16.4 months). The operative time was 65–180 min (average 124 min), and the blood loss was 20–200 ml (average 106 ml). All patients were treated with open reduction and internal fixation operation. According to the fracture characteristics, targeted reduction of the articular surface and restoration of the lower limb force line were performed. The fixed plate was placed anteriorly to fix the fracture fragment. The patients with simple medial plateau fracture were treated with anterior medial single plate (Fig. 1), the patients with simple lateral plateau fracture were treated with lateral plate (Fig. 2), and the patients with bilateral plateau fracture were treated with medial + lateral plates (Fig. 3). The posterior cruciate ligament injury was treated separately in the second stage. Menisci repair was performed in 8 patients, of which 5 were medial and 3 were lateral. For the patient with popliteal artery injury, popliteal artery exploration + saphenous vein transplantation + external fixation were performed firstly, and fracture treatment was performed at 2 weeks after the initial operation. For 2 patients with osteofascial compartment syndrome, fasciotomy + external fixation were performed firstly, then the wound was sutured 1 week after the initial operation, and fracture treatment was performed 3 weeks after the initial operation. For 2 patients with common peroneal nerve injury, no emergency surgery was performed, and common peroneal nerve exploration was performed during the fracture treatment operation. No common peroneal nerve rupture was seen. Fifteen patients received autogenous bone grafts, and the other patients underwent artificial bone or allogeneic bone grafts.

### Follow-up and functional evaluation

Evaluation of fracture reduction after surgery were as follows: anatomic reduction in 20 cases, good reduction in 5 cases (between 2 and 5 mm of articular surface collapse), and excellent fracture reduction rate of 100%. Internal fixation was as follows: 4 cases with simple lateral plate, 2 cases with simple medial plate, 15 cases with medial + lateral plates, 1 case with medial plate + lateral suture anchor, 1 case with medial plate + posterior screw, 1 case with lateral plate + fibular screw, and 1 case with medial and lateral plates + suture anchor. All fracture achieved bone healing at the last follow-up, and the fracture healing time was 3–6 months (average 3.3 months). Postoperative Rasmussen score was 18–29 (mean 24.9), and postoperative knee joint mobility was 90–130° (mean 118°). There were 3 patients who had PLC injury, and 1 patient had PCL injury repaired, because the joint could not achieve stability after bone reconstruction. Other 5 patients had MCL injury, and 1 patient had PCL injury who did not receive ligament repair. The Rasmussen score in no repaired patients was 24.2 compared with repaired patients which was 23.8 (*P* = 0.06), and the knee joint mobility in no repaired patients was 113.3° compared with repaired patients which was 118.8° (P = 0.54). The Rasmussen score in no ligament injury patients was 25.5 compared with ligament injury patients which was 24.0 (*P* = 0.51), and the knee joint mobility in no ligament injury patients was 119.3° compared with ligament injury patients which was 115.5°(*P* = 0.46). We used ROC curve (receiver operator characteristic curve) analysis to find the best cutoff value of pTSA degree change. In our results, 10° of pTSA change made the significant meaning. The area under curve was nearly 1 and *P* < 0.001. Vascular recanalization was good in the patient with popliteal artery injury, and no vascular embolism and limb ischemic necrosis occurred. Patients with compartment syndrome had no obvious muscle necrosis, and the function of the affected limbs recovered well (Table 2).

**Table 2:** Patients’ postoperative evaluation

No	Operation time	Bleeding volume	Fracture reduction	Internal fixation	Fracture healing time	Rasmussen score	Knee joint mobility	Complications
1	100	100	Anatomic reduction	Lateral plate	3	26	110	/
2	150	200	Anatomic reduction	Medial + lateral plates	6	25	110	/
3	130	100	Anatomic reduction	Medial + lateral plates	3	26	120	/
4	125	80	Anatomic reduction	Medial + lateral plates	3	27	120	/
5	130	100	Good reduction	Medial + lateral plates	3	24	110	/
6	140	150	Anatomic reduction	Medial + lateral plates	3	18	105	Peroneal nerve injury
7	155	150	Anatomic reduction	Medial + lateral plates	4	26	110	Superfacial infection
8	130	150	Good reduction	Medial + lateral plates	3	22	90	/
9	90	50	Anatomic reduction	Medial + lateral plates	3	29	130	/
10	100	80	Anatomic reduction	Medial plate + lateral suture anchor	3	23	125	/
11	65	20	Anatomic reduction	Lateral plate	3	29	130	/
12	105	100	Anatomic reduction	Lateral plate	3	26	120	Artery injury
13	120	100	Anatomic reduction	Medial + lateral plates	3	28	130	/
14	110	50	Anatomic reduction	Medial + lateral plates	3	19	115	Peroneal nerve injury
15	125	100	Anatomic reduction	Medial + lateral plates	3	23	100	Compartment syndrome
16	180	200	Anatomic reduction	Medial plate + posterior screw	5	23	105	/
17	130	120	Good reduction	Lateral plate + fibular screw	3	25	125	/
18	135	150	Anatomic reduction	Medial + lateral plates	3	24	120	/
19	130	120	Good reduction	Medial plate	3	24	120	/
20	115	100	Good reduction	Medial + lateral plates	3	23	115	/
21	140	150	Anatomic reduction	Medial and lateral plates + suture anchor	4	24	120	Compartment syndrome + superfacial infection
22	100	100	Anatomic reduction	Lateral plate	3	27	130	/
23	130	50	Anatomic reduction	Medial plate	4	26	125	/
24	120	60	Anatomic reduction	Medial + lateral plates	3	27	130	/
25	140	80	Anatomic reduction	Medial + lateral plates	3	28	130	/

### Complication

Two patients developed infections on the surface of the wound after surgery. The infection manifested as wound exudate around 2 weeks after surgery, healed after the wound dressing. During these two patients, one patient was complicated with osteofascial compartment syndrome, and the other patient was with comminuted three-column fractures suffered a long operation time. Two patients with common peroneal nerve injury had poor recovery with partial recovery of sensory function and no significant recovery of ankle dorsiflexion. They could walk with the aid of braces, but the gait was limited. There were no internal fixation failed, no postoperative fascial compartment syndrome, and no postoperative complications such as vascular and nerve injury occurred.

## Discussion

According to the injury mechanism, this type of fracture is currently considered to be caused by axial force with or without varus and valgus force when the knee joint is in the hyperextended position, which is easy to be accompanied by diagonal injury [9, 10]. In the previous literature, the tibial plateau pTSA angle has been used as the basis for imaging diagnosis. However, for fracture-dislocation type tibial plateau fractures, the pTSA always does not change, so we think it is more accurate to use “non-dislocated hyperextension tibial plateau fracture” to describe these hyperextension tibial plateau fracture which was mainly bone structure changes. According to the violence direction, hyperextension tibial plateau fractures are usually divided into simple hyperextension fractures, hyperextension varus fractures, and hyperextension valgus fractures [6]; according to the fracture line position, it can be divided into simple anterior compression fracture, anterior compression fracture with fracture line extending to the posterior cortical bone, and anterior compression fracture with posterior fracture fragment displacement [11]. The classification according to violence direction is more universal because it can guide treatment protocol. In non-dislocated hyperextension injury, the tibial plateau often causes anterior plateau fractures, which can cause forward instability, resulting in knee overextension. Without the fracture anatomically reduced, the knee joint cannot be located in the normal position when walking. Even small changes in the pTSA angle will increase the tension of the posterior cruciate ligament, causing the knee instability and affecting walking. Even if the bony structure reaches the standard for conservative treatment of tibial plateau fractures (articular surface collapse is less than 2 mm, varus is less than 5°, and the pTSA change is less than 10° [12, 13]; we still believe that this type of fracture requires active surgery to restore knee stability.

Such fractures are easily combined with soft tissue complications, such as compartment syndrome, popliteal vessel injury, and peroneal nerve injuries [14]. In our cases, there were 1 patient with popliteal artery injury, 2 patients with common peroneal nerve injury, and 2 patients with compartment syndrome, which had a higher incidence of vascular nerve injury than ordinary tibial plateau fractures [13]. Hyperextension varus injury causes compression fracture of the anterior medial plateau, and the posterior structure is extremely stretched, so it is easy to damage the popliteal artery and common peroneal nerve [15]. Two of our patients with common peroneal nerve injury both were hyperextension varus injury. For patients with vascular injury, surgical exploration must be carried out as soon as possible to restore the blood supply to save the limbs. For patients with common peroneal nerve injury, stretch injuries are generally considered. Neural exploration can be performed during fracture treatment surgery instead of emergency surgery, but the prognosis is often relatively poor.

For such fractures, the incidence of ligament soft tissue injury is high. The statistical incidence of our cases is 10 (10/25). These data are very similar to the 27% incidence reported in previous literature [16]. We routinely perform an MRI examination of the knee joint before surgery to determine the degree of damage to soft tissue ligaments and other structures. According to our statistics, the degree of articular surface collapse and the change of tibial pTSA can be used to judge whether there is damage to the posterior soft tissue structure partly. Patients with unilateral column fractures are more prone to “diagonal” injuries than patients with bilateral column fractures, and the patients with tibial plateau pTSA change greater than 10° were easier with ligament injury than the patients’ pTSA change less than 10°, which is helpful to make preoperative plan if MRI is performed unconditionally. Porrino J et al. [17] showed that the degree of ligament injury in knee joint was related to the severity of Schatzker type, and the higher the level of Schatzker type, the greater the possibility of ligament injury. Spiro AS et al. [18] showed that the compression degree of tibial plateau articular surface could reflect the injury of lateral meniscus and anterior cruciate ligament to a certain extent. The possibility of injury of lateral meniscus and anterior cruciate ligament increased by 15% and 18% respectively for every 1 mm of articular surface collapse, but there is no similar report in the previous literatures on the relationship between the degree of pTSA change and soft tissue injury. This study is the first to summarize the correlation between the two, but the sample size of our patients is small and there is the possibility of clinical bias, so it is necessary to expand the sample size to further confirm.

The fracture collapse of this type of injury is located in the front, so the approach should be slightly forward, and the surgical approaches should be decided according to the site of plateau involvement. For a single plateau fracture, the medial/lateral approach can be used to obtain satisfactory exposure. For a fracture involving the medial and lateral plateau or the total anterior plateau, both medial and lateral incisions often require for surgical treatment and this time should pay attention to the distance between skin bridges, generally more than 10 cm which will not cause skin healing problems. The anterior middle approach of the knee joint is also an option [19]. This method can fully expose the tibial plateau and intercondylar fossa. However, the soft tissue complication of this approach is more serious, which is easy to cause skin and soft tissue necrosis and infection. Therefore, we have not adopted this approach for treatment in our cases. For this type of fracture, the reduction is performed through the reverse injury mechanism. Generally, the knee joint is placed in the flexion position for reduction. The anatomical reduction is achieved by lifting the collapsed bone mass, restoring the articular surface and the lower limb force. After the fracture reduction, it is still necessary to judge whether soft tissue reconstruction is needed. In our cases, simple lateral plateau fractures with medial collateral ligament injury all achieved good knee stability after lateral plateau reduction, so no medial collateral ligament repair was performed. In patients with medial plateau fracture, there is often posterolateral structural instability after reduction of medial plateau fracture. Xie X et al. [20] reported a type of varus type fractures in which 51% of patients had avulsion of fibula head or 60% posterior tension failure, so we repaired the posterolateral structure with corresponding suture anchor or wire tension bands. Gonzalez LJ et al. [16] showed that bilateral hyperextension tibial plateau fractures had no significant difference in knee joint mobility and pain during follow-up compared with other types of plateau fractures, but the knee function score was poor. This suggests that we need to pay more attention to this type of fracture, and we need to adopt the “total knee joint repair” treatment concept to achieve satisfactory clinical prognosis.

## Conclusions

The main imaging characteristic of “non-dislocated hyperextension tibial plateau fracture” is the change of tibial plateau posterior slope angle. The injury of single anteromedial column/anterolateral column fracture is easy to combine with “diagonal” injury, and when the tPSA changes more than 10°, it is easy to be combined with ligament injury. By reducing the joint articular surface and lower limb force line, repairing the soft tissue structure, and reconstructing the knee joint stability, we can get satisfactory results.

## Data Availability

The datasets used and/or analyzed during the current study are available from the corresponding author on reasonable request.
